# Antimicrobial Effectiveness Testing of Resorbable Electrospun Fiber Matrix per United States Pharmacopeia (USP) <51>

**DOI:** 10.7759/cureus.50055

**Published:** 2023-12-06

**Authors:** Emily Sallade, Derayvia Grimes, Lily Jeng, Matthew R MacEwan

**Affiliations:** 1 Clinical Research, Acera Surgical, Inc., St. Louis, USA; 2 Research and Development, Acera Surgical, Inc., St. Louis, USA

**Keywords:** traumatic wound, surgical wound, contamination, non-healing wound, contaminated wound, wound healing, usp <51> antimicrobial effectiveness testing, electrospinning, synthetic electrospun fiber matrix, extracellular matrix

## Abstract

Contamination of surgical, traumatic, and chronic wounds with microorganisms presents a challenge to successful wound healing. In the present in vitro study, a synthetic electrospun fiber matrix (SEFM) cleared for use in the management of chronic, surgical, and traumatic wounds underwent USP (United States Pharmacopeia) <51> Antimicrobial Effectiveness Testing to determine its in vitro effectiveness against various microorganisms commonly found in non-healing wounds. The SEFM was tested in both sheet (s-SEFM) and micronized form (m-SEFM) against *Escherichia coli, Pseudomonas aeruginosa*, *Staphylococcus aureus*, *Aspergillus brasiliensis*, *Candida albicans,* *Proteus mirabilis*, and *Enterococcus faecalis*. Testing was performed per the USP <51> standard on days 7, 14, and 28. Both the s-SEFM and m-SEFM met the USP <51> acceptance criteria for all microorganisms. The results obtained for s-SEFM demonstrated >1-log_10_ reduction against* E. coli, S. aureus, P. aeruginosa, P. mirabilis, E. faecalis, *and* C. albicans *at day 7; >3-log_10_ reduction with no detection of these microbes at days 14 and 28, and no increase from initial inoculum at days 7, 14, and 28 against *A. brasiliensis.* The results obtained for m-SEFM demonstrated >3-log_10 _reduction with no detectable microorganisms at day 7. The results observed in this study indicate that the SEFM is effective in vitro at inhibiting bacterial and fungal growth and colonization per USP <51> testing.

## Introduction

Bacterial colonization of a wound represents one of the most common causes of delayed wound healing and commonly leads to health complications, such as active infection, abscess formation, wound dehiscence, and/or amputation [1-2]. Surgical site infections (SSIs) are among the most common healthcare-associated infection types [3]. Up to 5% of patients develop SSIs despite the peri-operative procedures in place to mitigate this risk, such as antibiotic prophylaxis, antimicrobial sutures, appropriate surgical site preparation, and sterile technique requirements in the operating room [3]. Certain procedures and patient populations are at increased risk of developing postoperative infections. Factors such as length of the procedure, patient age, vascularity, nutritional status, and comorbidities such as obesity and diabetes mellitus put patients at risk of delayed healing or infection [4]. Traumatic wounds tend to present with some degree of contamination due to the nature of the injury and thus may require surgical intervention to remove foreign materials and non-viable tissue [5]. The risk of infection in traumatic wounds can be reduced with appropriate debridement and cleansing, but despite these measures, deep SSIs can occur in roughly 6% of these patients at 30 days postoperatively, and as high as 13% at 90 days in lower extremity injuries [5]. Contaminants, including microorganisms, foreign bodies, debris, and biofilm, may vary depending on the wound type and can result in increased inflammation, less generation of granular tissue, and impeded re-epithelialization resulting in non-healing [1,6-7]. Considering the high risk of infection, which can result in poor healing outcomes, prophylactic management of surgical and traumatic wounds for infection control should be emphasized.

The predominant isolates found in wounds have been shown to be *Staphylococcus aureus, Pseudomonas aeruginosa, Proteus mirabilis, Escherichia coli, *and *Enterococcus faecalis *[1,6,8]. Studies have demonstrated that among gram-negative microorganisms, *P. aeruginosa, E. coli, *and *P. mirabilis *were the most represented species within wounds, while among gram-positive bacteria, *S. aureus* represented the most predominant species, followed by *E. faecalis* [1,6,8]. Gram-positive bacterial infections can be particularly difficult due to their drug resistance [9]. rDNA pyrosequencing of various wound bacterial microbiomes demonstrated high rates of polymicrobial contamination, revealing that 93% of wounds were contaminated with more than one species [10]. Fungal microorganisms can also lead to impaired wound healing [7]. Certain fungal species, such as *C. albicans*, are among the most common in the human microbiota, but overgrowth can lead to infection [7]. Additionally, *A. brasiliensis*, while ubiquitous in the environment, can result in mold-associated disease, especially within immunocompromised individuals [11]. Together, these seven microorganisms represent formidable impediments to successful wound healing.

Advanced matrices have been utilized in wound repair for decades [12]. These products may be cellular or acellular in nature and can be derived from either autologous, allogeneic, xenogeneic, or synthetic sources [12]. A synthetic, electrospun fiber matrix (SEFM) (Restrata®, Acera Surgical, Inc., St. Louis, MO) is a commercially available advanced matrix product [13] classified as a fully synthetic skin substitute by the Centers for Medicare and Medicaid Services. The SEFM is a soft, white, conformable, nonfriable, absorbable material available in both sheet form (s-SEFM) and in micronized form (m-SEFM) (MiniMatrix®, Acera Surgical, Inc., St. Louis, Mo) [13]. Both the s-SEFM and m-SEFM are Class II medical devices cleared by the U.S. Food and Drug Administration (FDA) through a 510 (k) process (K223725, K193583) when indicated for the management of wounds, including surgical, trauma, and full-thickness wounds [13]. The SEFM has demonstrated clinical efficacy in a number of both retrospective and prospective studies across varying wound etiologies [13-16]. This matrix is unique in both its composition and mechanism of action. The SEFM is composed of two bioresorbable synthetic polymers (polydioxanone and polyglactin 910), which are electrospun to resemble the scale and architecture of a native human extracellular matrix [13,14]. This engineered design provides a scaffold for cellular infiltration and vascularization without the use of biological products or added growth factors [13,14]. The porosity of the matrix and hydrolytic resorption rate are engineered to match the rate of new tissue formation [13,14]. As the SEFM resorbs, the porosity increases, permitting further cellular ingrowth and neovascularization [13,14]. During hydrolytic resorption of the SEFM, degradation products, including glycolic and lactic acid, are formed, and these acids can lower the local pH to create a mildly acidic microenvironment [13,14]. Prior studies have shown that local acidification may result in a bacteriostatic or bactericidal effect, yet few studies have examined this effect in the context of electrospun materials for human clinical use. It was therefore hypothesized that the SEFM would inhibit the growth and colonization of bacterial and fungal strains commonly found in non-healing wounds [17].

The present in vitro studies aimed to evaluate the efficacy of the SEFM in both sheet and micronized forms against a broad spectrum of microorganisms, including those most commonly found in wounds.

## Materials and methods

**Figure 1:**
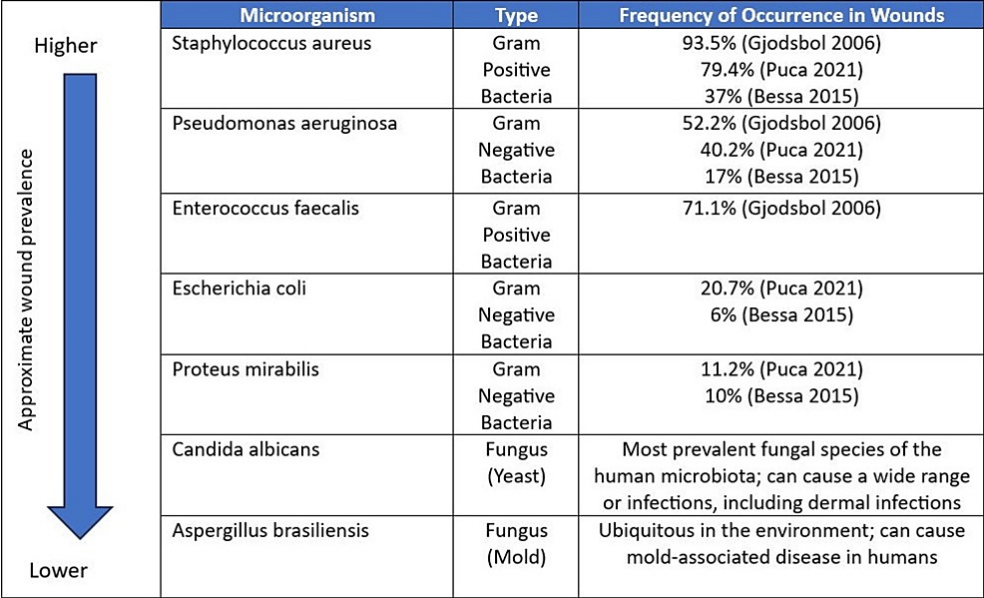
Frequently occurring microorganisms present in wounds. This figure is an original work of the authors.

Testing for the s-SEFM and m-SEFM were conducted separately. In both studies, testing was performed per USP <51> Antimicrobial Effectiveness Testing, a standardized test used to evaluate the in vitro antimicrobial effectiveness of materials [18]. The microorganisms selected for testing were *Escherichia coli *ATCC (American Type Culture Collection (ATCC ® Manassas, VA) 8739, *Pseudomonas aeruginosa *ATCC9027 (MicroBiologics, St. Cloud, MN), *Staphylococcus aureus *ATCC6538, *Aspergillus brasiliensis *ATCC16404, *Candida albicans *ATCC10231, *Proteus mirabilis *ATCC7002, and *Enterococcus faecalis *ATCC51575. These microorganisms were selected based on current literature documenting the most prevalent species found in open wounds (Figure 1).

The studies were conducted in accordance with the FDA's Good Laboratory Practice (GLP) for non‑clinical laboratory studies regulation, which is set forth in 21 CFR Part 58 [19].

Preparation

Preparation of Microbial Test Cultures

The following cultures were prepared for both studies. For all bacteria (*E. coli, P. aeruginosa, S. aureus, *and *P. mirabilis*), the culture was suspended in 10 mL sterile Tryptic Soy Broth (TSB) prior to initiating the neutralization verification and the test. The suspension was incubated at 36±1°C for 18-24 hours. For* A. brasiliensis*, culture from the most recent working stock culture was streaked to sabouraud dextrose agar (SDA) or equivalent slants and incubated at 30±2°C for 6-10 days. For *C. albicans*, culture from the most recent working stock culture was streaked to potato dextrose agar (PDA) prior to initiating neutralization verification, and the test plate was incubated at 30±2°C for 44-52 hours. 

Preparation of Test Inocula

For all bacterial cultures, each culture was centrifuged for at least 10 minutes, and the cell pellet was suspended in sterile phosphate-buffered saline (PBS) in an appropriate volume (6.0 mL-11.0 mL) to achieve a concentration estimated to ~1 X 10^9^ CFU (colony forming units)/mL. For *A. brasiliensis*, surface growth was washed from the incubated SDA slants using sterile PBS supplemented with 0.1% Triton X 100 (v/v). For *C. albicans*, individual colonies were suspended in sterile PBS to achieve a concentration estimated at ~1 X 10^9^ CFU/mL.

Preparation of Test Substances for Inoculation

The test substances were prepared by soaking the s-SEFM in physiological saline (0.9% sodium chloride) in a ratio of 6 cm^2^ s-SEFM to 1.0 mL physiological saline, and the m-SEFM in a ratio of 0.2 g to 1.0 mL physiological saline. The mixtures were incubated at 50 ± 2 °C for 72 ± 2 hours, and the supernatants were removed for testing. The supernatant containing s-SEFM was prepared for testing by adding 10 mL of supernatant to each of the seven sterile conical tubes. The supernatant containing m-SEFM was prepared by adding 1 mL of supernatant to each of the seven sterile conical tubes. 

Inoculation

Each vessel containing the test substance was inoculated with 0.050 mL of each test inocula preparation. Each tube was vortex mixed. All tubes were incubated at 15-30°C for 28 days, with viability sampling performed at 7, 14, and 28 days. 

Determination of Initial Inocula Concentrations

10 mL of sterile PBS was inoculated with an identical volume of each test system inocula (0.050 mL) used for the inoculation of the s-SEFM test substances. For m-SEFM, 1mL of sterile PBS was inoculated. The control tubes were vortex mixed and a serial 1:10 dilution series was performed in sterile PBS. The appropriate dilutions were plated in duplicate to the associated growth-supporting agar.

Viability sampling 

For the s-SEFM test, 1.0 mL of inoculated s-SEFM test substance was harvested and added to 9.0 mL of neutralization broth. For the m-SEFM test, 0.1 mL of the inoculated test substance was harvested and added to 9.9 ml of neutralization broth. The vessels were vortex-mixed, and serial 1:10 dilutions were performed in sterile PBS. Dilutions were plated in duplicate by standard dilution and plate count techniques.

Neutralization validation and recoverability comparison 

A neutralization control was performed per test microorganism per test substance. One milliliter of s-SEFM test substance was added to seven neutralizer broth tubes, containing 9.0 mL of Dey/Engley broth, to represent the “Neutralization Test” tubes. A 1.0 mL volume of sterile PBS was added to seven separate and identical volume neutralizer tubes to represent the “Neutralization Control” tubes. Each tube was inoculated with 0.065 mL, for all strains except *A. brasiliensis,* or 0.050 mL for *A. brasiliensis*, aliquot of dilute test microorganism suspension obtained by serial dilution in sterile PBS.

Next, 0.1 mL of m-SEFM test substance was added to seven neutralizer broth tubes which contained 9.9 mL of Dey/Engley broth. A 0.1 mL volume of PBS was added to the seven separate and identical volume neutralizer tubes to represent the “Neutralization Control” tubes. Each tube was inoculated with a 0.050 mL aliquot of the dilute microorganism suspension. Test and Control Tubes were vortex mixed, and a 1.0 mL aliquot was plated in duplicate to the appropriate growth-supporting agar.

Controls

Media Sterility Control

At each viability sampling interval, including the initial enumeration of the starting inocula, an aliquot of each liquid media/reagent (PBS, Dey/Engley broth, etc.) used in the study was plated individually to each agar media used in the study. At each viability sampling interval, including the initial enumeration of the starting inocula, one plate containing only the agar medium used in this study was incubated to determine agar sterility. 

Media Viability and Culture Purity Control

At each time a new test culture was generated and used, a loop full of each test microorganism culture was struck to the appropriate growth agar to achieve isolated colonies to confirm purity and viability.

Incubation of enumeration and control plates 

*E. coli, P. aeruginosa, S. aureus, P. mirabilis*, and *E. faecalis *enumeration plates were incubated at 36 ± 1°C for 48 ± 4 hours. *A. brasiliensis* and *C. albicans* enumeration plates were incubated at 30 ± 2°C for 48 ± 4 hours. All media sterility controls were incubated at 30 ± 2°C for 48 ± 4 hours. All viability/purity controls are incubated at the appropriate temperature and time for the test system.

Calculations

**Figure 2:**
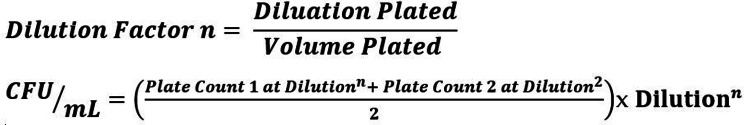
Calculation used to determine microorganism concentration at enumeration steps CFU: Colony forming units.

The calculation below was used to determine the concentration of each test microorganism at the enumeration steps (Figure 2).

Bacteria and yeast dilations demonstrating colony counts > 300 colonies were noted as too numerous to count (TNTC). Only results demonstrating adequate colony counts (≤ 300 colonies) were used for calculations. Mold dilutions demonstrating colony counts >150 colonies were noted as TNTC. Only results demonstrating adequate colony counts (≤150 colonies) were used for calculations.

**Figure 3:**
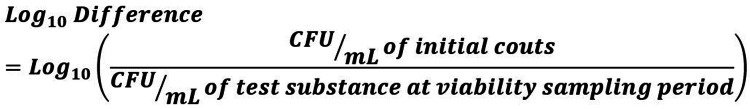
The calculation used to determine the difference of each test microorganism at the enumeration compared to the initial counts CFU: Colony forming units.

The following calculation was used to determine the Log_10_ difference of each test microorganism at the previously mentioned enumeration steps compared to the initial counts (Figure 3).

**Figure 4:**
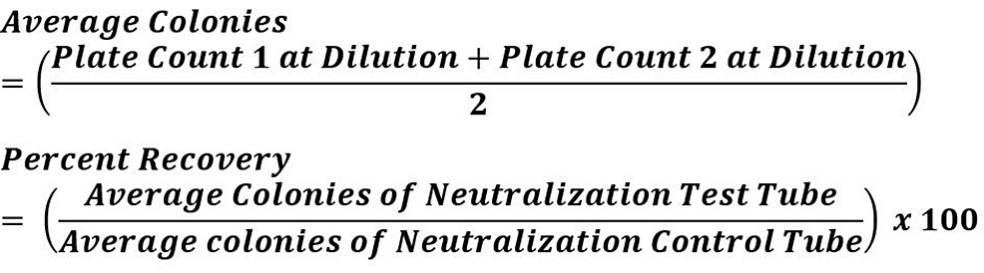
The calculation used to determine the percent recovery of each microorganism for Neutralization Verification

The following calculation was used to determine the percent recovery of each microorganism for the Neutralization Verification (Figure 4).

Acceptance criteria for Category 1 products per USP <51> Antimicrobial Effectiveness Testing

The SEFM, a USP <51> Category 1 product in both sheet and micronized form, had the following acceptance criteria per the standard. The criteria for demonstrating antimicrobial effectiveness for bacteria are: no less than 1.0 Log_10_ reduction from the initial inoculum count at 7 days, not less than 3.0 Log_10 _reduction from the initial inoculum count at 14 days, and no increase from the 14 day’s viability sampling interval count at 28 days.

The criteria for demonstrating antimicrobial effectiveness against yeast and molds are: no increase from the initial inoculum count at 7, 14, and 28 days, with no increase defined as no greater than a 0.5 log increase from the initial inoculum count.

## Results

The SEFM in both sheet and micronized form met passing criteria per USP <51> Antimicrobial Effectiveness Testing against all seven microorganisms tested: *E. coli, S. aureus, P. aeruginosa, P. mirabilis, E. faecalis, C. albicans, and A. brasiliensis*.

The s-SEFM findings showed >1-log_10_ reduction against *E. coli, S. aureus, P. aeruginosa, P. mirabilis, E. faecalis,* and *C. albicans *at the day 7 sampling timepoint, and >3-Log_10_ reduction to no detectable colonies of those microbes by days 14 and 28. The results also demonstrated no increase of *A. brasiliensis *from the initial inoculum at all time points (Table 1).

**Table 1:** Results of USP <51> Antimicrobial Effectiveness Testing for SEFM sheets The limit of detection for this assay was 5 CFU/mL. No increase is defined as no greater than a 0.5 Log increase from the initial inoculum count. USP = United States Pharmacopeia; SEFM = synthetic electrospun fiber matrix; s-SEFM = sheet synthetic electrospun fiber matrix; CFU = colony forming units.

Test Substance	Contact Time	Data Description	Test Microorganism						
			E. coli	P. aeruginosa	S. aureus	P. mirabilis	E. faecalis	A. brasiliensis	C. albicans
s-SEFM	Time Zero	CFU/mL	6.85E +05	8.95E+05	1.73E+05	1.50E+06	5.90E+05	2.75E+05	5.25E +05
	Day 7	CFU/mL	<5.00E+00	<5.00E+00	<5.00E+00	<5.00E +00	<5.00E+00	3.45E+05	2.00E+01
		Log_10_ Reduction	>5.14	>5.25	>4.54	>5.48	>5.07	-0.10	4.42
	Day 14	CFU/mL	<5.00E+00	<5.00E+00	<5.00E+00	<5.00E+00	<5.00E+00	3.90E+05	<5.00E+00
		Log_10_ Reduction	>5.14	>5.25	>4.54	>5.48	>5.07	-0.15	>5.02
	Day 28	CFU/mL	<5.00E+00	<5.00E+00	<5.00E+00	<5.00E+00	<5.00E+00	2.75E+05	<5.00E+00
		Log_10_ Reduction	>5.14	>5.25	>4.54	>5.48	>5.07	0.00	>5.02
USP <51> Antimicrobial Effectiveness Testing of the S-SEFM + test microorganism			Passed	Passed	Passed	Passed	Passed	Passed	Passed

The m-SEFM findings showed a >3-log_10_ reduction against all microorganisms tested at all sampling time points, with a reduction to no detectable colonies of these microbes at day 7 (Table 2).

**Table 2:** Results of USP <51> Antimicrobial Effectiveness Testing for micronized SEFM The limit of detection for this assay was 50 CFU/mL. Values observed below this limit are presented as <5.00E+01 in the table above. USP = United States Pharmacopeia; SEFM = synthetic electrospun fiber matrix; m-SEFM = micronized synthetic electrospun fiber matrix; CFU = colony forming units.

Test Substance	Contact Time	Data Description	Test Microorganism						
			E. coli	P. aeruginosa	S. aureus	P. mirabilis	E. faecalis	A. brasiliensis	C. albicans
m-SEFM	Time Zero	CFU/mL	4.75E+05	8.90E+05	4.50E+05	9.55E+05	3.10E+05	4.45E+-5	4.90E+05
	Day 7	CFU/mL	<5.00E+01	<5.00E+01	<5.00E+01	<5.00E+01	<5.00E+01	<5.00E+01	<5.00E+01
		Log_10_ Reduction	>3.98	>4.25	>3.95	>4.28	>3.79	>3.95	>3.99
	Day 14	CFU/mL	<5.00E+01	<5.00E+01	<5.00E+01	<5.00E+01	<5.00E+01	<5.00E+01	<5.00E+01
		Log_10_ Reduction	>3.98	>4.25	>3.95	>4.28	>3.79	>3.95	>3.99
	Day 28	CFU/mL	<5.00E+01	<5.00E+01	<5.00E+01	<5.00E+01	<5.00E+01	<5.00E+01	<5.00E+01
		Log_10_ Reduction	>3.98	>4.25	>3.95	>4.28	>3.79	>3.95	>3.99
USP <51> Antimicrobial Effectiveness Testing of the M-SEFM + test microorganism			Passed	Passed	Passed	Passed	Passed	Passed	Passed

## Discussion

Reduction of microbial contamination from a wound bed is a critical step necessary to facilitate effective healing. In this laboratory study, the SEFM in both sheet and micronized form was found to be effective per USP <51> Antimicrobial Effectiveness Testing against a broad range of microorganisms (bacteria, yeast, and other fungi) commonly found within wounds. The s-SEFM results demonstrated >3-Log_10 _reduction with no detection of six microorganisms by day 14 (*P. aeruginosa, S. aureus, E. coli, E. faecalis, P. mirabilis,* and *C. albicans*) and inhibition of growth of a 7^th^ microorganism (*A. brasiliensis*) during the course of the 28-day study. m-SEFM results demonstrated >3-Log_10_ with no detection of any microorganisms by day 7.

The USP <51> standard provides a well-accepted test method for evaluating the effectiveness of a material against a broad spectrum of microorganisms [18]. The seven microorganisms selected in this study were chosen because of their known pathogenicity, representing a comprehensive range of microbes typically found in the wound environment [1,6-8,11-12,20]. Gjødsbøl et al. (2006) conducted a retrospective clinical study on 46 patients with chronic leg ulcers [6]. A total of 342 samples were taken from the 46 ulcers. A total of 37 bacterial species were identified in the ulcer samples. The most common bacterial species detected in chronic ulcers was *S. aureus*, which was isolated at least once during the study period in 93.5% of the ulcers [6]. The next most prevalent species found were *E. faecalis* (71.1%) and *P. aeruginosa* (52.2%) [6]. In Puca et al. (2021) study, 239 wound samples were collected from 239 patients, with *S. aureus* (79.4%) *P. aeruginosa* (40.2%), *E. coli* (20.7%), and *P. mirabilis* (11.2%) being were the most represented species [8]. Bessa et al. (2015) investigated 312 wound samples collected from 213 patients in which a total of 28 different microbial species were isolated; the most common bacterial species detected was *S. aureus* (37%), followed by *P. aeruginosa* (17%), *P. mirabilis *(10%), and *E. coli* (6%) [1].

Microbial contamination or colonization of a wound often results in delayed healing and poor clinical outcomes [1,6,8,20]. Acute wounds, such as those resulting from surgical procedures or traumatic injury, can progress to non-healing or chronic wounds if an infection develops during the healing process [20]. Given the significant morbidity, mortality, and cost associated with SSIs and other wound infections, prophylactic infection management is vital to improving clinical outcomes [21].

USP <51> testing was performed separately on the SEFM in both sheet and micronized form. While both met passing criteria, the m-SEFM demonstrated a slightly more rapid reduction of microorganisms than the s-SEFM. The authors theorize this effect is observed due to the increased surface area to volume ratio within m-SEFM. Particulates with higher surface areas may hydrolyze more rapidly, eliciting a more robust change in local pH [22]. Clinically, m-SEFM is designed to offer a simple and convenient treatment for surgical or traumatic wound management, particularly in wounds with difficult topography or cavities. The micronized matrix is beneficial in this setting, as it can be dispersed within the wound bed to effectively reach difficult wound areas and support successful wound healing. Traumatic wounds often require surgical debridement to adequately address wound bed contamination, and have an increased risk of infection [23]. 

The composition of the SEFM is hypothesized to elicit the effect on bacterial and fungal strains observed in the current study. The degradation and resorption process of the SEFM may lower the pH of the surrounding environment and create a mildly acidic microenvironment. Acidic environments have demonstrated inhibition of bacterial and fungal growth in clinically relevant ways [24]. For example, a decrease in the pH of the skin is important for supporting the presence of protective microflora and minimizing unwanted fungal overgrowth [24]. Additionally, as the pH of the local microenvironment decreases, a reduction in bacterial integrity due to disruption of the lipids in bacterial cell membranes may be observed [25].

Within surgical wound management, products exist to serve either as an advanced wound healing matrix to encourage cellular ingrowth and tissue formation (i.e., bovine collagen matrices) or as a wound dressing to provide coverage over an open wound and aid in managing infection (e.g., silver dressings) [26]. Advanced wound healing matrices, such as acellular matrices, undergo a denaturation process to remove cellular material [27]. This process can damage the natural extracellular structure and limit cellular proliferation [27]. Additionally, xenograft acellular dermal matrices are unable to resist infection alone without antimicrobial additives [28]. Collagen-based materials, which are prevalent in both advanced wound healing matrices and wound dressings, are subject to premature enzymatic degradation resulting in a limited therapeutic window [14]. Collagen-based materials alone cannot offer antimicrobial resistance, and available products lack the adequate native structure needed to protect against microorganisms [17]. Silver is a commonly used, non-biologic additive utilized in antimicrobial wound dressings. Silver is cytotoxic to many bacterial and fungal strains, however, it is also toxic to fibroblasts in high concentrations, and as such, should be utilized with care to ensure positive clinical outcomes [29]. Biologic antimicrobial dressings benefit from the use of synthetic materials, which demonstrate resistance to enzymatic degradation and maintain wound moisture [13]. While wound dressings demonstrate antimicrobial effects and provide a physical barrier, they do not demonstrate the structural design needed to encourage tissue ingrowth [17]. The critical distinction is that wound dressings primarily provide coverage from external factors, and in some cases, maintain a moist wound environment, while advanced wound healing modalities, such as wound healing matrices, offer comparable advantages while providing a framework for tissue regeneration. The 3-dimensional structure of the SEFM encourages cellular ingrowth and proliferation while maintaining a moist wound environment. The results of the present study suggest the SEFM may serve both purposes without the inclusion of any added active agents. To the authors’ knowledge, this is the only product to possess these unique properties [20,30].

It should be noted that testing was conducted in a highly controlled laboratory setting, and the present data has not been replicated in human clinical settings. Human clinical settings introduce additional biological factors that cannot be accounted for in vitro. Only a select number of microorganisms were included in the testing, and the impact of bacterial resistance was not accounted for. Considering these factors, additional testing should be considered to draw conclusions on the effect of the SEFM on other bacterial and fungal strains, including bacterial strains that have developed resistance. Additionally, clinical studies should be considered to further assess the passing results documented in the USP <51> Antimicrobial Effectiveness Testing and how this translates into a real-world setting. We did not study the extrapolation of these results to clinical performance.

## Conclusions

SEFM, both in sheet and micronized forms, underwent antimicrobial effectiveness testing per <USP-51> and met passing criteria for each of the seven microorganisms tested, including *P. aeruginosa, S. aureus, E. coli, E. faecalis, P. mirabilis, C. albicans,* and *A. brasiliensis.* Although the in vitro testing does not necessarily extrapolate to clinical performance, these microorganisms are among the most prevalent in traumatic, surgical, and non-healing wounds. When these microorganisms are left unmanaged, they may often lead to systemic infection and other complications. To the author's knowledge, SEFM is the only wound healing matrix to pass USP <51> Antimicrobial Effectiveness Testing without the use of additives.
